# Irreducible inguinoscrotal hernia containing cecum and inflamed appendix: an unusual variant of Amyand’s hernia managed with Lichtenstein mesh repair – a case report

**DOI:** 10.1097/RC9.0000000000001097

**Published:** 2026-10-12

**Authors:** Subhash C. Mandal, Aakash Giri, Dinesh Nalbo, Ranjit Kumar Yadav

**Affiliations:** Department of Surgery, BP Koirala Institute of Health Sciences, Dharan, Nepal

**Keywords:** Amyand’s hernia, appendectomy, cecal herniation, Lichtenstein mesh repair, Losanoff–Basson classification, resource-limited setting

## Abstract

**Introduction:**

Amyand’s hernia occurs in approximately 0.1–1.7% of all inguinal hernias. Concurrent cecal herniation is a rare variant, reported only in isolated case reports and not documented as a distinct category in large systematic reviews. Management in resource-limited settings, particularly in patients with metabolic comorbidities, is sparsely reported in the literature.

**Presentation of case:**

A 46-year-old man with chronic alcohol use presented with a 6-month history of irreducible right inguinoscrotal swelling. Examination revealed a 12 × 8 cm pyriform mass. Laboratory findings indicated alcohol-related hepatic dysfunction and thrombocytopenia. Surgical planning was guided by ultrasonography in the absence of computed tomography (CT). Intraoperative findings confirmed a Losanoff–Basson Type 2 Amyand’s hernia with concurrent cecal herniation; an appendectomy and Lichtenstein mesh repair were performed. Postoperative alcohol withdrawal necessitated ICU admission.

**Discussion:**

Concurrent cecal herniation with Amyand’s hernia can be identified clinically and by ultrasonography when CT is unavailable. Mesh repair can be performed in Losanoff–Basson Type 2 cases without peritoneal contamination, as supported by emerging literature. A multidisciplinary approach can manage alcohol-related metabolic comorbidity.

**Conclusion:**

Amyand’s hernia with concomitant cecal herniation is a rare surgical case. Individualized decision-making regarding appendectomy and mesh use and systematic management of alcohol-related comorbidities are essential for favorable outcomes in resource-limited settings.

## Introduction

Amyand’s hernia is a rare type of inguinal hernia in which the vermiform appendix is contained within the inguinal hernia sac. It was first described in 1735 by Claudius Amyand, who performed the first successful appendectomy in such a case. The incidence of Amyand’s hernia accounts for approximately 0.1–1.7% of all inguinal hernias, with male predominance and right-sided predilection^[^1,2^]^. The condition is usually diagnosed intraoperatively because its clinical features resemble those of a typical inguinal hernia. Although ultrasonography (USG) and computed tomography (CT) scans help in preoperative diagnosis, confirmation is often achieved intraoperatively^[^3,4^]^. Left-sided cases are rare and are usually associated with anatomical variations such as a mobile cecum, intestinal malrotation, or situs inversus^[^5^]^.

HIGHLIGHTS

- Concurrent cecal herniation with an Amyand’s hernia is an exceptionally rare surgical finding.
- Ultrasonography can aid in preoperative assessment when CT is unavailable in resource-limited settings.
- Intraoperative findings confirmed a Losanoff–Basson Type 2 Amyand’s hernia with a viable cecum and an inflamed appendix.
- An appendectomy with Lichtenstein mesh repair was successfully performed without peritoneal contamination.
- Mesh use should be individualized according to contamination status and patient-specific factors.

Cecal herniation alongside the appendix is even less common, as the cecum is normally fixed retroperitoneally. Its presence usually reflects a mobile cecum combined with a wide or deep inguinal ring^[^6^]^, and it is clinically relevant because the herniated bowel may be at increased risk of obstruction, strangulation, or perforation^[^7^]^.

The use of prosthetic mesh remains debated for Losanoff–Basson Type 2; the original classification recommends primary repair without mesh if appendicitis is present, whereas recent studies have questioned its use in the absence of gross contamination^[^1,8,9^]^. This case report describes the diagnosis and management of an unusual variant of Amyand’s hernia with cecal involvement in a resource-limited setting without a CT scan, which was complicated by alcohol-related hepatic dysfunction and thrombocytopenia, and discusses the rationale for mesh use in this context.

## Case presentation

A 46-year-old male patient presented to the emergency department with swelling in the right inguinoscrotal region for the past 6 months. The swelling was initially small, soft, and easily reducible, increasing in size during coughing or straining and would reduce spontaneously. The swelling gradually increased in size and extended into the scrotal region over several months. He had a 15-year history of chronic alcohol consumption, with no prior diagnosis of liver disease or hematologic disorder, but showed alcohol-related hepatic dysfunction and thrombocytopenia at presentation, as shown in Table 1.

**Table 1:** Preoperative and immediate postoperative investigations.

SN	Investigation	Preoperative	Postoperative
1	Hemoglobin	13.1 g/dL	11.9 g/dL
2	Total leucocyte count	6530 cells/µL	4780 cells/µL
3	Platelet	60 × 10^3^/µL	84 × 10^3^/µL
4	PT	14 seconds	16 seconds
5	INR	1	1.13
6	Urea	26.07 mg/dL	15.29 mg/dL
7	Creatinine	1.10 mg/dL	0.70 mg/dL
8	Sodium	136.37 mEq/L	136.99 mEq/L
9	Potassium	3.83 mEq/L	3.61 mEq/L
10	Amylase	66.81 U/L	Not repeated
11	Lipase	77.94 U/L	Not repeated
12	Total/conjugated bilirubin	2.76/0.60 mg/dL	1.87/0.62 mg/dL
13	ALT/AST/ALP	49.72/91.12/96.09 U/L	47.93/94.23/96.79 U/L
14	Total protein/Albumin	7.57/4.52 g/dL	6.00/3.96 g/dL

Five days before presentation, the swelling became increasingly painful and irreducible, prompting urgent medical attention. He reported constipation but no history of nausea, vomiting, abdominal pain, abdominal distension, urinary symptoms, or fever, with no history or examination findings suggestive of appendicitis or perforation preoperatively.

On general examination, the patient was conscious, cooperative, and in distress due to pain but hemodynamically stable. On physical examination, there was a pyriform-shaped, tender, non-reducible, soft-in-consistency, non-pulsatile swelling measuring 12 × 8 cm in the right inguinoscrotal region. The transillumination test was negative. The right testis was palpable but not clearly delineated from the hernia mass; the left testis was normal in size and consistency. No inguinal or femoral lymphadenopathy was noted. Penile examination was unremarkable. Digital rectal examination was normal. Bowel sounds were audible over the swelling on auscultation. Vital signs were: temperature 98.7°F, pulse 100 beats per minute, blood pressure 137/85 mm Hg, respiratory rate 20 breaths per minute, and oxygen saturation 92% on room air.

USG of the inguinoscrotal regions showed herniated bowel loops with echogenic content extending into the right scrotal sac, without free fluid. Color Doppler demonstrated preserved vascularity. Laboratory investigations revealed thrombocytopenia, hyperbilirubinemia, and elevated hepatic transaminases consistent with alcohol-induced liver dysfunction, as presented in Table 1. A CT scan was not performed due to limited availability. Based on clinical and ultrasonographic findings, a working diagnosis of irreducible inguinoscrotal hernia was made without evidence of strangulation. Surgical exploration was therefore planned on this basis.

An approximately 6 cm incision was made in the right inguinal region, centered over the midpoint of the inguinal ligament. The external oblique aponeurosis was incised to enter the inguinal canal. A right-sided indirect inguinal hernia was identified. The hernia sac was dissected free from the spermatic cord structures, and the vas deferens and testicular vessels were preserved. On opening the sac, the cecum with the vermiform appendix was identified within it (Fig. 1). The cecum was viable, the internal ring patulous, and the appendix edematous without perforation or peritoneal contamination, consistent with Type 2 of the Losanoff–Basson classification^[^3^]^. No fecalith was identified. An appendectomy was performed; the cecum was subsequently reduced, and the Lichtenstein tension-free mesh technique was used, with a 15 × 7 cm polypropylene mesh placed over the posterior wall of the inguinal canal and fixed with 2-0 polypropylene sutures on a round-body needle. Surgery was performed under general anesthesia lasting approximately 90 minutes, with an estimated blood loss of 60 mL. The surgical wound was closed in anatomical layers. No drains were placed. Perioperative antibiotics, IV ceftriaxone and ornidazole, were given per standard institutional protocol for appendectomy.

**Figure 1.**
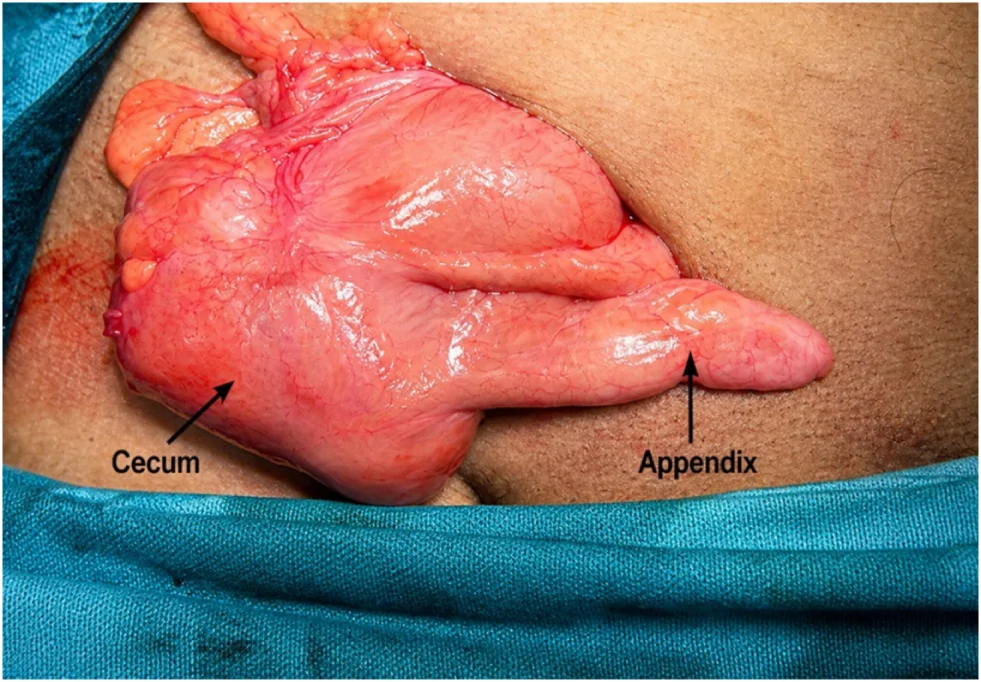
Intraoperative findings showing the contents of the indirect inguinal hernia sac: cecum and inflamed appendix.

Postoperative alcohol withdrawal syndrome was anticipated, and the patient was admitted to the ICU for 3 days and managed with a fixed-schedule diazepam and haloperidol regimen; intravenous thiamine was administered under psychiatric consultation. On the fourth postoperative day, he was transferred to the surgical ward and discharged on the sixth day following clinical stabilization. The histopathological report confirmed acute appendicitis with transmural inflammation. At the outpatient review 4 weeks post-discharge, the wound had healed satisfactorily with no clinical evidence of hernia recurrence.

## Discussion

Concurrent cecal herniation within an Amyand’s hernia sac is an uncommon anatomical variant that has not been specifically mentioned, even in systematic reviews of hundreds of cases^[^1^]^, with only isolated reports describing this finding^[^6,7,10,11^]^. The present case is distinguished by an emergency presentation without CT imaging, significant alcohol comorbidity, and mesh repair in Losanoff–Basson Type 2 disease, which required justification beyond the standard classification framework.

In this case, cecal herniation is attributable to a mobile cecum, an embryological variant involving incomplete fusion to the lateral peritoneal wall^[^6^]^, combined with a wide, deep inguinal ring identified intraoperatively. Most frequently, cecal herniation is identified as a predisposing anatomical factor in published cases of ileocecal herniation within Amyand’s hernia^[^10,11^]^. The 6-month duration likely explains the appendiceal inflammation due to prolonged compression within the sac rather than an intraluminal cause of typical appendicitis, as proposed by Rojas *et al*^[^12^]^.

The management depends on the degree of appendiceal involvement, the surgeon’s expertise, and patient-specific considerations. A classification system introduced by Losanoff and Basson (Table 2) in 2007^[^3^]^, and later modified by Singal *et al* (Table 3)^[^13^]^, guides surgical decision-making^[^9^]^. The present case corresponds to Type 2 of the Losanoff–Basson classification.

**Table 2:** Losanoff and Basson classification of Amyand’s hernia.

Classification	Description	Management
Type 1	Normal appendix within an inguinal hernia	Reduction of hernia and mesh repair
Type 2	Acute appendicitis within an inguinal hernia without abdominal sepsis	Appendectomy with primary hernia repair without mesh
Type 3	Acute appendicitis within an inguinal hernia with abdominal and/or peritoneal wall sepsis	Laparotomy with appendectomy and primary hernia repair without mesh
Type 4	Acute appendicitis within an inguinal hernia with associated intra-abdominal disease	Same as Type 3, along with the management of the associated disease

**Table 3:** Modified (Rikki/Singal) classification of Amyand’s hernia.

Classification	Description	Management
Type 1	Normal appendix within an inguinal hernia	Reduction of hernia and mesh repair
Type 2	Acute appendicitis within an inguinal hernia without abdominal sepsis	Appendectomy with primary repair without mesh
Type 3	Acute appendicitis within an inguinal hernia with abdominal and/or peritoneal wall sepsis	Laparotomy with appendectomy and primary repair without mesh
Type 4	Acute appendicitis within an inguinal hernia with associated intra-abdominal pathology	Same as Type 3, with additional management of the associated condition
Type 5a	Normal appendix within an incisional hernia	Reduction of hernia with primary repair, including mesh
Type 5b	Acute appendicitis within an incisional hernia without peritonitis	Appendectomy with primary fascial closure, no mesh repair
Type 5c	Acute appendicitis within an incisional hernia with peritonitis or abdominal wall sepsis or related to prior surgery	Same as Type 4

A few published cases suggest the range of presentations. Sun *et al* reported a giant right inguinoscrotal hernia containing an inflamed appendix, abscess, cecum, terminal ileum, and descending colon, managed with appendectomy and mesh repair, with no recurrence at 4-week follow-up, which was consistent with our approach^[^7^]^. In contrast, Jha *et al* described management by emergency laparotomy for a perforated cecum within an obstructed left-sided hernia, irrespective of classification type^[^10^]^, reflecting that contamination status influenced the approach. Similarly, Tcheutchoua Soh *et al* reported two strangulated hernias containing cecum and inflamed appendix, managed by appendectomy and mesh placement^[^11^]^. All these reports suggest that contamination status is the key determinant of mesh use.

Whether to perform an appendectomy at the time of hernia repair remains debated. Some authors advocate routine appendectomy to prevent potential future complications^[^3^]^, while others recommend preserving a normal appendix to minimize infection risk^[^1,9^]^. Given the appendiceal inflammation confirmed intraoperatively, appendectomy was considered appropriate. In our patient, the absence of perforation, peritoneal contamination, or purulent exudate supported mesh placement, consistent with a 2025 systematic review of 123 patients with Amyand’s hernia, which found that mesh was used in a substantial minority of cases even in appendicitis without gross contamination^[^14^]^. Similarly, a related review supported the use of mesh in Type 2 after appendectomy, reserving tissue repair for grossly contaminated fields^[^2^]^. This reinforces prioritizing contamination status rather than classification type alone^[^1,8,9^]^.

This case adds to the small body of literature suggesting that, in the absence of contamination, appendectomy with Lichtenstein mesh repair may be reasonable in selected patients with cecal herniation, consistent with systematic reviews reporting the safety of mesh in clean or minimally contaminated Type 2 cases^[^1,8,9^]^. Although conclusions cannot be drawn from a single case, the decision to use mesh should be individualized. Ultrasound may serve as first-line imaging to guide surgical planning when CT is unavailable. In this case, alcohol withdrawal was anticipated given a 15-year history, and proactive ICU admission with psychiatric input contributed to an uncomplicated resolution. Surgeons in resource-limited settings should have a high index of suspicion for unusual hernia sac contents and be prepared to make individualized operative decisions.

## Limitations

As a single case report, this study cannot establish standard management recommendations, and the use of mesh should be interpreted cautiously. The 4-week follow-up is insufficient to assess recurrence or mesh-related outcomes. In addition, the diagnosis was based on clinical assessment and ultrasonographic findings without CT confirmation, limiting anatomical characterization.

## Conclusion

Amyand’s hernia with concomitant cecal herniation is an uncommon surgical finding. In this case, appendectomy with Lichtenstein repair was performed in the absence of contamination, with a favorable short-term outcome. The intraoperative decision was based on contamination status rather than classification type alone, alongside systematic management of alcohol-related comorbidities. Longer follow-up would help to clarify the recurrence and mesh-related complications.

## Data Availability

Not applicable.
